# A Brief Exposure to Leftward Prismatic Adaptation Enhances the Representation of the Ipsilateral, Right Visual Field in the Right Inferior Parietal Lobule

**DOI:** 10.1523/ENEURO.0310-17.2017

**Published:** 2017-09-27

**Authors:** Sonia Crottaz-Herbette, Eleonora Fornari, Isabel Tissieres, Stephanie Clarke

**Affiliations:** 1 Neuropsychology and Neurorehabilitation Service, Centre Hospitalier Universitaire Vaudois (CHUV), and University of Lausanne, 1011 Lausanne, Switzerland; 2 Centre d'Imagerie Biomédicale, Department of Radiology, CHUV, and University of Lausanne, 1011 Lausanne, Switzerland

**Keywords:** fMRI, inferior parietal lobule, prismatic adaptation, visual field

## Abstract

A brief exposure to rightward prismatic adaptation (PA) was shown to shift visual field representation within the inferior parietal lobule (IPL) from the right to the left hemisphere. This change in hemispheric dominance could be interpreted as (1) a general effect of discrepancy in visuomotor alignment caused by PA or (2) a direction-specific effect of rightward PA. To test these hypotheses, we compared the effects of rightward and leftward PA on visual representation in normal human subjects. Three groups of normal subjects underwent an fMRI evaluation using a simple visual detection task before and after brief PA exposure using leftward- or rightward-deviating prisms or no prisms (L-PA, R-PA, neutral groups). A two-way ANOVA group × session revealed a significant interaction suggesting that PA-induced modulation is direction specific. *Post hoc* analysis showed that L-PA enhanced the representation of the right visual field within the right IPL. Thus, a brief exposure to L-PA enhanced right hemispheric dominance within the ventral attentional system, which is the opposite effect of the previously described shift in hemispheric dominance following R-PA. The direction-specific effects suggest that the underlying neural mechanisms involve the fine-tuning of specific visuomotor networks. The enhancement of right hemispheric dominance following L-PA offers a parsimonious explanation for neglect-like symptoms described previously in normal subjects.

## Significance Statement

Leftward-deviating prisms (L-PA) increased the representation of the right visual field within the right inferior parietal lobule (IPL). This enhancement of the right hemispheric dominance within the ventral attentional system contradicts the dominance shift, from right to left hemisphere, which is induced by rightward-deviating prisms (R-PA). Thus, the PA-induced modulation of hemispheric dominance within the ventral attentional system is sensitive to the direction of prismatic deviation and is likely to depend on fine-tuning of specific visuomotor networks. The overemphasis of right visual field representation within the (right) ventral attentional system offers a parsimonious explanation for neglect-like effects following L-PA.

## Introduction

Prismatic adaptation (PA) consists of a brief session during which subjects point to targets under visual control while wearing goggles with prisms that deviate the visual field to the right or to the left. First pointings are characterized by errors that disappear after 10–15 trials. The adaptation is typically measured once the prisms are removed by the so-called “aftereffect” that corresponds to the pointing errors opposite to the deviation and that reflects the prism-induced sensorimotor realignment (Weiner et al., 1983). Adaptation to rightward-deviating prisms (R-PA) yields a systematic leftward deviation of visuomotor and proprioceptive responses, whereas adaptation to leftward-deviating prisms (L-PA) yields a systematic rightward deviation (Rossetti et al., 1998; Redding et al., 2005; Jacquin-Courtois et al., 2013).

The neural processes underlying ongoing PA have been studied in normal subjects during different stages of PA. These studies showed primary activation within the parieto-temporal cortex and the cerebellum, suggesting a visual and proprioceptive spatial realignment during L-PA (Luauté et al., 2009; Chapman et al., 2010) and R-PA (Danckert et al., 2008); alternating L-PA and R-PA was used in an early study and provided evidence for the involvement of parietal cortex in adaptation (Clower et al., 1996). The effects after the adaptation have been investigated in normal subjects using rightward deviating prisms (Crottaz-Herbette et al., 2014). By comparing task-related activations acquired pre and post-PA, this study showed that R-PA bilaterally modulated the activation in the inferior parietal lobule (IPL) during visual target detection by increasing the representation of left, central, and right visual fields in the left IPL and by decreasing the representation of right and central visual fields in the right IPL. Thus, R-PA shifted hemispheric dominance for visuospatial representation within the ventral attentional system from the right to the left hemisphere; this shift is most likely one of the key mechanisms which underlies therapeutic effect of R-PA in neglect (Clarke and Crottaz-Herbette, 2016).

This rapid change in hemispheric dominance could be interpreted in two different ways. First, it may be induced by any discrepancy in sensorimotor realignment, possibly by uncovering pre-existing bilateral visual representations within the left IPL (de Haan et al., 2015) or by tapping into the left-dominant motor attentional system (Rushworth et al., 2001, 2003). If this is the case, then adaptations to leftward or rightward prisms should lead to similar modulations of the ventral attentional system with an increased activation of the left IPL and a decreased activation of the right IPL during a visual detection task after both adaptations. Second, the change in hemispheric dominance may be specific to the direction of PA, suggesting that fine-tuning of visuospatial representations in response to specific visuomotor adaptation plays a critical role. In the case of direction specificity, L-PA could be expected to yield the opposite effect to R-PA, namely, to increase activation in the right IPL in response to ipsilateral, right targets. If present, the effect of L-PA may offer a highly interesting therapeutic option for the treatment of attentional disorders, which can occur in left hemispheric stroke (Murakami et al., 2014). To test the two hypotheses, we compared the effects of L-PA and R-PA on visual representation. The current study involved three groups of normal subjects who underwent functional MRI during a simple visual detection task before and after a brief adaptation session wearing leftward- or rightward-deviating prisms or plain glasses (L-PA, R-PA, neutral groups).

## Materials and Methods

### Participants

Forty-two participants were included in this study, with 14 participants (seven men, mean age = 24.1, SD = 3.0 years) undergoing L-PA, 14 participants (seven men, mean age = 26.0 years, SD = 5.0 years) undergoing R-PA, and 14 participants (seven men, mean age = 25.8 years, SD = 5.1 years) in the control group (neutral). A one-way ANOVA comparing the mean age between the three groups did not show a significant difference between the groups (*F*_(2,39)_ = 0.85, *p* = 0.44). All participants were right handed (Oldfield, 1971) and had a normal or corrected-to-normal vision. None of the subjects had a neurological or psychiatric illnesses. All participants gave written informed consent according to procedures approved by the Ethics Committee of the Faculty of Biology and Medicine, University of Lausanne.

### Experimental design

The same procedure was used for the L-PA, R-PA, and neutral groups, comprising two MRI blocks that were separated by an intervention using visuomotor adaptation. MRI blocks consisted of anatomical sequences (only before the adaptation) and event-related fMRI acquisitions (before and after the adaptation). The R-PA and neutral groups did two other tasks that were analyzed elsewhere (Crottaz-Herbette et al., 2014). The delay between the adaptation and the detection task was the same for the three groups.

#### Visual detection task

During the fMRI acquisitions, all participants had to press the response button when they detected a large white star on black background. These visual stimuli were presented for 500 ms in three different locations: in the midsagittal plane, at 20° to the right or 20° to the left. The locations were pseudorandomized and each location was presented 20 times. The interevent intervals were jittered, between 1 and 20 s with steps of 1 s. During this task, participants were asked to fixate on a central fixation point. Participants responded by pressing a button with their right hand as soon as they detected the visual stimulus. The tasks were programmed using the software E-Prime (Psychology Software Tools). The duration of the task was 6 min 44 s.

#### Visuomotor adaptation

The visuomotor adaptation was performed outside the scanner and consisted of pointing with the one index finger to visual targets presented 14° to the left or to the right of the midsagittal plane. The prisms (www.optiquepeter.com) deviated the visual field 10° to the left for the L-PA group and to the right for the R-PA group (Rossetti et al., 1998; Redding et al., 2005; Rode et al., 2006); goggles without deviation were used for the neutral group. During the pointing movements, participants in the R-PA and neutral groups used their right index finger whereas participants in the L-PA group used their left index finger. With the exception of the hand used during the adaptation, the procedure for PA, including the positioning of the participants, was similar across our three groups.

The choice of the left hand for pointing in the L-PA group was motivated by putative clinical implications. If L-PA enhances right hemispheric dominance within the ventral attentional system, as postulated in our hypothesis, it may offer an interesting therapeutic approach for attentional disorders in left hemispheric stroke (which is often associated with motor deficits of the right upper limb). Each participant’s head was immobilized in a head rest and the first two thirds of the pointing trajectories were hidden from his/her view. The visuomotor adaptation involved 3 min of pointing movements. The pointing was paced by the experimenter, who indicated verbally which of the two points should be targeted next. To avoid automatic pointing, the intertrial interval varied (1.0–1.5 s) and the order of targets was pseudorandomized. The total number of pointing movements was on average 150 (range = 145–155). The time for pointing was kept constant across subjects, as was the time between the two fMRI sessions.

During the first trials, participants showed initial errors in the direction of the prisms’ deviation, and then they all pointed correctly to the targets. Immediately after the goggles were removed, the aftereffect was assessed by asking the participants to look at one of the visual targets and then to close their eyes and to reach for the target with the index finger used during the adaptation. A similar procedure was used twice for the left target and twice for the right target in a pseudorandom order; the number of measures was limited in order to minimize de-adaptation before the second fMRI session. For each participant and each target position, we put a mark on the table where the participant pointed, and we measured, in mm, the deviation between the pointing and the actual target, with positive values representing a deviation to the right of the targets and negative values representing a deviation to the left of the targets. We averaged the two pointings for each target location. A mixed design ANOVA with group (R-PA, L-PA, neutral) as a between-subjects factor and side of target (left, right) as a within-subjects factor was conducted on these data.

### Data acquisition

Imaging acquisitions, structural MRI and event-related fMRI were conducted at the Lemanic Biomedical Imaging Center (Centre d’Imagerie Biomédicale) in the Centre Hospitalier Universitaire Vaudois, Lausanne on a 3T Siemens Magnetom Trio scanner with a 32-channel head-coil. A single-shot echo planar imaging gradient echo sequence (repetition time = 2 s; flip angle = 90°; echo time = 30 ms; number of slices = 32; voxel size = 3 × 3 × 3 mm; 10% gap) was used for fMRI acquisitions. A total of 32 slices were acquired in the AC-PC plane in a sequential ascending order and covered the whole head volume. For each participant, a high-resolution T1-weighted 3D gradient-echo sequence was acquired (160 slices, voxel size = 1 × 1 × 1 mm). We put padding around each participant’s head to prevent head movements in the coil.

### Data analysis

Behavioral performances (reaction time and number of correct responses) recorded during the task were analyzed with a mixed design ANOVA with group (R-PA, L-PA, neutral) as the between-subjects factor and session (1, 2) as the within-subjects factor. The software Statistical Parametric Mapping (SPM8, Wellcome Department of Cognitive Neurology, London, United Kingdom) was used to process imaging data. For the functional acquisition, a motion correction was performed by applying a 6-parameter rigid-body transformation minimizing the difference between each image and the first scan. These realigned images were co-registered with the participants’ anatomic images and then normalized to the Montreal Neurological Institute (MNI) template using a twelve parameters affine transformation. Finally, these images were resliced to obtain a 2 × 2 × 2 mm voxel size and spatially smoothed using an isotropic Gaussian kernel of 6-mm FWHM to increase signal-to-noise ratio.

For each participant, the general linear model, as implemented in SPM8 software (http://www.fil.ion.ucl.ac.uk/spm/software/spm8/), was used for the first level statistics. The parameters of the realignment were included in the model as regressors. For all participants, contrasts of interests were specified for both sessions. The maps generated from these contrasts were used as the second-level (group-level) statistics based on the random field theory. All group analyses were restricted to voxels with the probability of belonging to gray matter greater than 50%, as defined in the a priori template available in SPM.

Statistical analyses on the activation maps were conducted on a general mixed design ANOVA that included the factors group (R-PA, L-PA, neutral) as the between-subjects factor and session (1, 2) and stimulus position (left, center, right) as the within-subjects factors. From this general ANOVA, the first analysis was on the interaction between the three factors (group × stimulus position × session) to determine the effects of our factors globally. Then, the interaction between the factors group and session was analyzed to determine the relationship between these two factors independent of the stimulus positions. The generated statistical maps of activation for these interactions were set at a threshold of *p* < 0.05 and a cluster extent of *k* > 100 (above the expected number of voxels per cluster as automatically calculated by SPM). The effects of each intervention were further investigated by directly comparing session 1 to session 2 (*post hoc t* tests) for each stimulus position and each group separately. The generated statistical maps of activation for these *t* tests were set at a threshold of *p* < 0.05 and a cluster extent of *k* > 150 (above the expected number of voxels per cluster as automatically calculated by SPM).

## Results

### Aftereffects of the visuomotor adaptation

The aftereffects of PA occurring after the removal of the prismatic goggles were assessed as pointing errors to the right or left of the actual target (expressed in positive and negative values, respectively). For the L-PA group, the pointing errors were always to the right of the left and right targets; the means of the pointing errors were +5.1 ± 2.4 cm for the left target and +5.9 ± 2.4 mm for the right target. For the R-PA group, the pointing errors were always to the left of the left and right targets. For this group, the means of the pointing errors were −66 ± 16 mm (mean ± SD) for the left target and −5.6 ± 1.9 mm for the right target. For the neutral group, pointing errors were to the right or to the left of the targets; mean pointing errors were +7.0 ± 1.1 cm for the left target and +6 ± 8 mm for the right target. A two-way mixed design ANOVA with group (L-PA, R-PA, neutral) as the between-subjects factor and side of target (left, right) as the within-subjects factor revealed a significant main effect of group (*F*_(2,39)_ = 314.9; *p* < 0.001) but no significant effect for the side of the target or interaction. The aftereffects were globally larger for the L-PA and R-PA than for the neutral group, with the R-PA group showing errors to the left of the targets and the L-PA group showing errors to the right of the targets.

### Behavioral results of the visual detection task

For accuracy (Table 1), an ANOVA including the factors groups (L-PA, R-PA, neutral), sessions (1, 2) and stimulus positions (left, center, right) did not show a significant effect. For the reaction times (Table 1), the ANOVA including the factors group (L-PA, R-PA, neutral), session (1, 2), and stimulus position (left, center, right) showed only one significant main effect for the factor stimulus position (*F*_(2,38)_ = 14.73, *p* < 0.01), with the subjects being globally faster for the central position.

**Table 1.** Average accuracy (mean ± SEM; top) and average reaction times (bottom) for the visual detection task for the L-PA, R-PA, and neutral groups for both sessions (1 and 2) and for all stimulus positions (left, central, and right targets)

	Left targets		Central targets		Right targets	
Session	1	2	1	2	1	2
	Accuracy (%)					
L-PA	99.64 ± 0.36	98.93 ± 1.07	98.93 ± 0.57	99.29 ± 0.49	99.64 ± 0.36	100.00 ± 0.00
R-PA	98.21 ± 1.00	99.29 ± 0.49	100.00 ± 0.00	99.29 ± 0.71	96.79 ± 1.62	99.64 ± 0.36
Neutral	100.00 ± 0.00	98.21 ± 1.79	99.64 ± 0.36	99.29 ± 0.49	99.64 ± 0.36	98.57 ± 1.10
Reaction time (ms)						
L-PA	388 ± 24	399 ± 17	383 ± 24	385 ± 17	379 ± 23	403 ± 19
R-PA	408 ± 15	416 ± 19	397 ± 15	403 ± 15	401 ± 13	414 ± 15
Neutral	375 ± 10	404 ± 14	360 ± 10	383 ± 14	365 ± 9	396 ± 10

### Modulation of activation patterns by interventions

**Figure 1.**
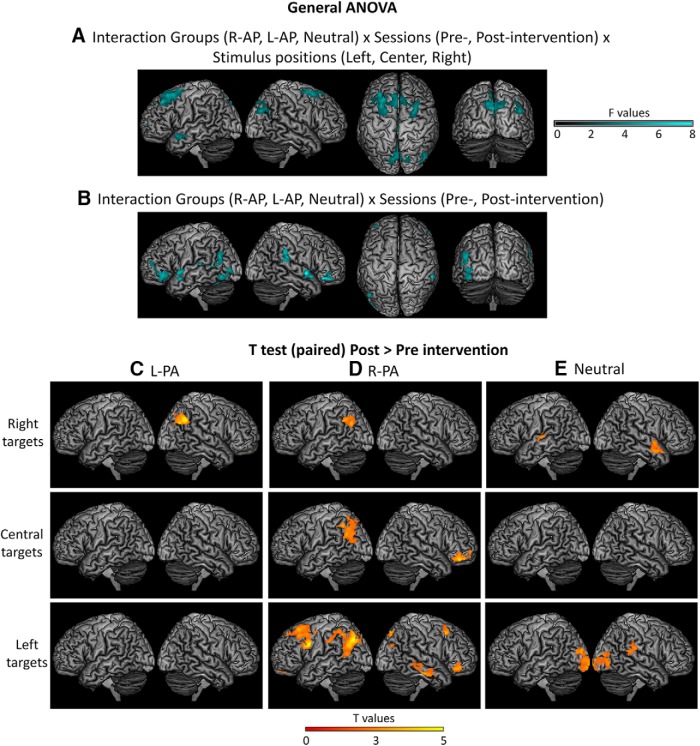
Surface renderings of the brain activation showing significant activation in the general mixed design ANOVA for the interaction between all three factors, group as the between-subjects factor and session and stimulus position as the within-subjects factors (***A***); and for the interaction between the factors group and session (***B***). ***C–E***, Surface renderings of *post hoc* paired *t* tests (post > pre-intervention) for the L-PA (***C***), R-PA (***D***), and neutral groups (***E***) for each stimulus position separately. All maps are set at a threshold of *p* < 0.05 and *k* > 100 for the interactions and *k* > 150 for the *t* tests.

The overall modulations were analyzed with a mixed design ANOVA with group (L-PA, R-PA, neutral) as the between-subjects factor and session (1, 2) and stimulus position (left, center, right) as the within-subjects factors. The triple interaction between the factors group, session, and stimulus position yielded a significant effect in the right angular gyrus, the left anterior superior and the middle temporal gyri and bilaterally in the superior (medial) parietal regions, the precuneus, medial and middle frontal gyri, SMA and the middle cingulate areas (Fig. 1*A*). The interaction between the factors group and session yielded a significant effect on the left hemisphere in the angular gyrus, the middle temporal gyrus and the middle occipital gyrus, on the right hemisphere in the supramarginal gyrus, and bilaterally in the superior temporal gyrus and the orbito-frontal cortex (Fig. 1*B*). These results indicate that the direction of prismatic deviation impacts the PA-induced modulation of activity within the left and the right IPL.

To gain insight into the direction-specific changes of the PA intervention, the effects were analyzed separately for each of the three intervention groups and stimulus position with paired *t* tests comparing activation pre- and postintervention. L-PA enhanced the response to right visual targets within the ipsilateral, right angular gyrus (Fig. 1*C*). R-PA enhanced the response to right, central, and left targets within the left IPL as described previously (Crottaz-Herbette et al., 2014), as well as in parts of the prefrontal and temporal cortexes for the central and right targets (Fig. 1*D*). Exposure to plain goggles increased the response to right targets bilaterally in the superior temporal gyrus and to left targets within the right supramarginal gyrus and bilaterally within the occipital cortex (Fig. 1*E*). Thus, there is a striking but opposing effect of PA depending on the direction of prismatic deviation. L-PA enhanced right hemispheric dominance within the ventral attentional system by increasing the representation of the right visual field within the ipsilateral, right IPL. R-PA shifted this hemispheric dominance from the right to the left IPL by increasing the representation of right, central, and left visual field within the left IPL (see also Crottaz-Herbette et al., 2014; Clarke and Crottaz-Herbette, 2016).

### Direction-specific effects of PA on hemispheric dominance within the ventral attentional system

IPL is classically subdivided into angular and supramarginal gyri, each of which comprises several subdivisions defined by cytoarchitectonic and connectivity criteria (Caspers et al., 2008; Mars et al., 2011). The effects which we report here involved mostly the angular and less so the supramarginal gyrus. The right angular gyrus showed a significant interaction between the factors group × session × stimulus position, which was driven by a strong increase in activation by right targets following L-PA (Fig. 1*A*,*C*). The left angular gyrus showed a significant interaction between the factors group × session, which was driven by a strong increase in activation by left, central, and right targets following R-PA (Fig. 1*B*,*D*; Crottaz-Herbette et al., 2014). In both hemispheres these clusters were within the cytoarchitectonic areas PGa and PGp of the angular gyrus (Caspers et al., 2008), known for their role in redirecting of visuospatial attention (Mort et al., 2003; Thiel et al., 2004). The supramarginal gyrus was highlighted on the right side by a significant interaction between the factors group × session, which appeared to be driven by an increase in activation in the control condition (Fig. 1*B*,*E*). This part of the supramarginal gyrus corresponds to the cytoarchitectonic areas PF and PFt (Caspers et al., 2008), which plays a role in visuomotor coordination (Binkofski et al., 1999; Frey et al., 2005; Grol et al., 2007). In summary, PA appears to affect the attentional module within the angular gyrus: L-PA increases the representation of right targets on the right side, whereas R-PA increases the representation of left, central, and right targets on the left side. The effect in right supramarginal gyrus appears to be driven by the control condition and may represent a modulation of visuomotor coordination.

**Figure 2.**
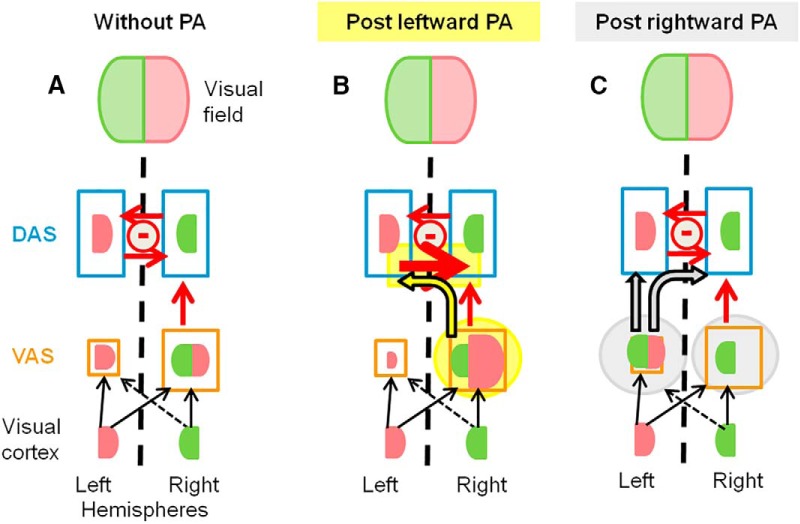
Schematic representation of the dorsal and ventral attentional systems (DAS, VAS, outlined in blue and orange, respectively), the visual areas, and their interactions (based on Koch et al., 2008; Corbetta and Shulman, 2011). Situations without PA (***A***) as well as after L-PA (***B***) and R-PA (***C***) are represented. L-PA-induced changes are highlighted in yellow: enhancement of right visual field representation in the right VAS (as reported in our current findings) and the increased inhibition from left to right DAS (resulting from change in respective excitability as in Schintu et al., 2016). R-PA-induced changes (***C***) are highlighted in gray (based on Crottaz-Herbette et al., 2014 and discussed in Clarke and Crottaz-Herbette, 2016).

As reported in a previous study, a brief exposure to R-PA increased the representation of the left, central, and right visual fields in the left IPL and shifted the hemispheric dominance within the ventral attentional system from the right to the left hemisphere (Crottaz-Herbette et al., 2014). This shift in hemispheric dominance offers a parsimonious explanation for behavioral effects of R-PA observed both in normal subjects and neglect patients (Clarke and Crottaz-Herbette, 2016). Our new results contrast with this effect as we found that a brief exposure to L-PA increases the representation of the right visual field in the right IPL, enhancing the right hemispheric dominance within the ventral attentional system (Fig. 2). This overemphasis of the right visual field within the (right-dominant) ventral attentional system offers an explanation for the behavioral effects of L-PA reported in several previous studies, including neglect-like performance. It also offers insight into the putative neural mechanisms that underlie the effect of L-PA.

## Discussion

### Behavioral effects of leftward PA

#### Neglect-like performance in normal subjects

Several studies in normal subjects have shown that L-PA induces neglect-like performance in some, but not all visuospatial tests (Michel, 2016). L-PA yielded a rightward bias on the perceptual variant of the line bisection task (Colent et al., 2000), including striking similarities with neglect symptoms, such as effect of line length and modulation of the rightward deviation by the position of the lines (Michel et al., 2003). This rightward bias in perceptual line bisection is long-lasting yet fluctuating, suggesting that the visuospatial shift needs time to build up (Schintu et al., 2014). L-PA also induced a rightward shift in visual midpoint judgments occurring both in peri- and extrapersonal spaces (Berberovic and Mattingley, 2003).

In the present study L-PA did not induce a lateral bias in the target detection task performed during the fMRI acquisition. The use of more complex tasks during the fMRI acquisition would be of interest in further studies for two reasons. First, more difficult detection tasks would allow us to assess a putative lateral bias in performance, possibly a neglect-like effect. It is to be noted, however, that in several studies L-PA failed to yield behavioral effects with the Posner paradigm (Morris et al., 2004; Bultitude et al., 2013a).This lack of behavioral effects contrasts with the results of event-related potentials to different components of the endogenous variants of the Posner task, which revealed attentional asymmetries that were reminiscent of neglect (Martín-Arévalo et al., 2016). With L-PA, but not with R-PA or neutral goggles, two measures stood out. The L-PA induced reduction of the N1 amplitude elicited by the cue was greater for leftward than rightward cues, suggesting an L-PA-induced asymmetry in attentional orienting. The L-PA-induced reduction of the P1 amplitude was greater for the invalidly cued left than right target, suggesting an asymmetry in attentional disengagement. Second, the use of bisection tasks, which have been shown to be modulated by L-PA (Michel and Cruz, 2015; Striemer et al., 2016), may help to explore the effect of L-PA beyond that on the ventral attentional system.

Our results offer a parsimonious explanation for neglect-like performance described above.

L-PA overemphasizes the responsiveness of the right IPL to stimuli presented within the right visual field. This stronger representation of the right visual field within the right-dominant ventral attentional system may facilitate the access of right stimuli to the dorsal system and drive the left dorsal attentional system more forcefully. An overactive left dorsal attentional system is bound to create a right attentional bias in behavioral tasks. In addition, it may increase the interhemispheric inhibition of the contralateral, right dorsal system and decrease its activity (Koch et al., 2011). This interpretation is supported by a recent study that has indeed demonstrated that L-PA increased the excitability of the parietal circuitry in the left and decreased it in the right hemisphere (Schintu et al., 2016).

##### *Modulation of global* vs. *local processing bias by L-PA*

Tasks that implicate attention to global vs. local features of stimuli rely on complex cortical networks (Fink et al., 1996, 1997). Although sustained attention to either level was shown to activate a right hemispheric temporo-parieto-prefrontal network, directing attention to global aspects highlighted specifically the role of the right lingual gyrus while attending to local aspects activated the left inferior occipital cortex. Performance in tasks such as Navon figures, with incongruent global and local features, are characterized in normal subjects by greater interference from global rather than local features. L-PA was shown to reduce the global processing bias (Bultitude and Woods, 2010). A later study using different paradigms, the rod-and-frame illusion and the simultaneous-tilt illusion, demonstrated that L-PA enhanced local processing bias (Reed and Dassonville, 2014). Thus, in normal subjects, L-PA shifted the processing bias from global to local features, as often found in neglect (Robertson et al., 1988; Marshall and Halligan, 1995). Our results offer only a partial explanation for these findings. After L-PA, the increased activation to ipsilateral targets within the right ventral attentional system (shown here) and the ensuing enhanced activity within the left dorsal attentional system (Schintu et al., 2016) may change the encoding within the left early-stage visual areas, including the inferior occipital cortex, and may thus favor the processing of local features.

#### Visuospatial remapping

Spatial remapping ensures the integration of visual information as gaze moves across a scene, resulting in a stable representation of the visual environment despite constantly changing retinal images. It depends critically on the right posterior parietal cortex (Heide et al., 1995; van Koningsbruggen et al., 2010). Using the double-step saccade paradigm, Bultitude and colleagues (Bultitude et al., 2013b) have shown that L-PA impairs spatial remapping in the left visual field. The authors proposed that the temporary realignment of spatial representations with L-PA altered right hemispheric remapping processes. Our results demonstrated right hemispheric remapping within the (right) ventral attentional system, but it concerns the right and not left visual space.

#### Behavioral effects of rightward PA

In normal subjects R-PA appears to yield behavioral effects only rarely. R-PA increase the speed of reflexive reorienting from invalid cues on the left to targets on the right side in a subgroup of subjects, who had large cueing effects before R-PA; no effect was reported on voluntary reorienting (Striemer et al., 2006). Another study found rightward shift in visual midpoint judgment in extrapersonal, but not in peripersonal space (Berberovic and Mattingley, 2003). A third study investigated spatial remapping with a double-step saccade paradigm (Bultitude et al., 2013b). R-PA affected oculomotor performance, most likely by low-level adaptation aftereffects, but did not yield any spatial remapping. The explanation for these three observations in terms of the shift of hemispheric dominance of the ventral attentional system from the right to the left hemisphere, which is induced by R-PA, were discussed in a recent review (Clarke and Crottaz-Herbette, 2016).

### Putative mechanisms of leftward PA

The effect of L-PA relies most likely on several functional systems, as suggested by a series of studies. Spatial realignment during the actual adaptation to prisms was shown to involve the parieto-temporal cortex and the cerebellum (Luauté et al., 2009; Chapman et al., 2010), with a critical contribution of the latter (Panico et al., 2016). At the level of the posterior parietal and primary motor cortices L-PA was found to induce hemispheric-specific changes in excitability: an increase in motor evoked potentials in the left and a decrease in the right hemisphere (Schintu et al., 2016). Here, we show that L-PA enhances the representation of the right visual field within the right IPL.

**Figure 3.**
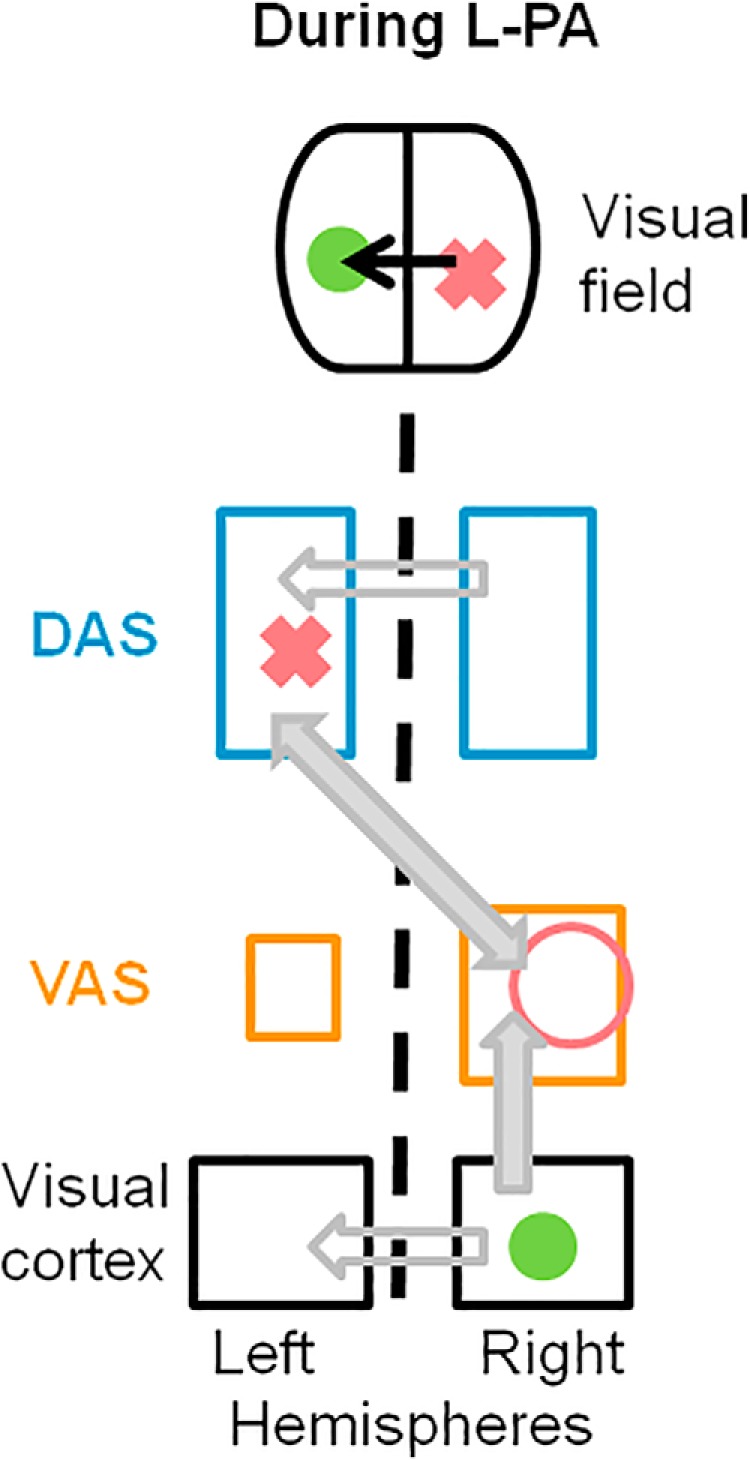
Schematic representation summarizing putative neural mechanisms that underlie the effects of L-PA. Same conventions as in Figure 2. The pink cross designates the actual position of the target and highlights the contralateral DAS, which is involved in pointing towards this target position. The green circle designates the position of the same target as perceived within the (left) visual space when wearing leftward deviating prisms, its representation in the right visual cortex and DAS. The pink circle highlights the L-PA-enhanced representation of the (right) visual field within the (right) DAS. Exposure to L-PA is likely to strengthen the link between the representation of the perceived target within the (right) visual cortex and the left DAS. The most likely link involves heterotopic interhemispheric connections between the (right) VAS and left DAS (full gray arrow). The homotopic interhemispheric connections between visual areas and those between DAS are unlikely to contribute (outlined gray arrows).

Taken together, the above quoted evidence suggests neural mechanisms which may underlie the effect of L-PA, and provides ground for new hypotheses and further studies. While the subject is wearing leftward-deviating prisms, targets appear to the left of their actual position. In Figure 3, we represent a simplified situation where the target is in the right visual field near the vertical meridian and L-PA shifts it into the left visual field so that the target activates the corresponding left visual field representation within the retinotopically organized visual areas of the right hemisphere. To point successfully towards the target, the movement has to be directed towards the actual site within the right hemispace; attention-driven movements towards the right hemispace are represented in the left superior parietal lobule (Leonards et al., 2000; Corbetta et al., 2002; Silver and Kastner, 2009). Thus, successful adaptation to leftward deviating prisms can be expected to involve several steps, including a modulation of salience of particular spatial representations within each hemisphere. Learning to associate a target which appears on the left side with a pointing movement oriented towards the right space is very likely to result in the strengthening of the link between the left visual field representations in the right occipital cortex and the dorsal attentional system in the left hemisphere. This link can be mediated by several pathways. First, the most likely pathway proceeds from visual areas in the right hemisphere to the (right) ventral attentional system and then via an interhemispheric connection to the left dorsal attentional system. Such heterotopic-crossed connections can be monosynaptic, as demonstrated histologically in the human occipito-parieto-temporal cortex (Di Virgilio and Clarke, 1997). The key observation of our study, namely, the reorganization within the (right) ventral attentional system, further supports this interpretation. Second, it is very unlikely that a functional link between the representations of the perceived and the actual position occur at the level of early-stage visual areas since the interhemispheric connections between these areas concern only a narrow part of the cortex along the representation of the vertical meridian (Clarke and Miklossy, 1990) and the intrahemispheric connections are retinotopically organized (Clarke, 1994). Third, the link is also unlikely to be mediated by afferents from the right to the left dorsal attentional system. A recent study has shown that right-to-left connections are lessened following L-PA, most likely as a result of an increase in parietal excitability in the left and a decrease in the right hemisphere (Schintu et al., 2016).

## Conclusion

L-PA increased the representation of the right visual field within the right IPL. This enhancement of the right hemispheric dominance within the ventral attentional system contrasts with the dominance shift, from right to left hemisphere, which is induced by R-PA (Crottaz-Herbette et al., 2014). Thus, the PA-induced modulation of hemispheric dominance within the ventral attentional system is sensitive to the direction of the prismatic deviation and is likely to depend on fine-tuning of specific visuomotor networks.

The overemphasis of the right visual field representation within the (right) ventral attentional system offers a parsimonious explanation of neglect-like effects following L-PA. It is bound to more forcefully drive the left dorsal attentional system, creating an attentional bias towards the right space. The underlying neural mechanisms most likely involve a strengthened link between the (right) ventral attentional system and the left dorsal attentional system.

The effect of L-PA, which we report in this study, is likely to be of considerable interest for the rehabilitation of attentional deficit in left hemispheric stroke. These deficits are frequent and often preclude the return to work and/or driving (Murakami et al., 2014). They may be the result of the re-organization which takes place within the intact hemisphere after unilateral focal lesions (Adriani et al., 2003). We have shown here that adaptation to left-deviating prisms by means of left-hand pointing enhances right hemispheric dominance within the ventral attentional system and may thus constitute a very useful therapeutic intervention in left hemispheric stroke.

## Synthesis

Reviewing Editor: Bradley Postle, University of Wisconsin Decisions are customarily a result of the Reviewing Editor and the peer reviewers coming together and discussing their recommendations until a consensus is reached. When revisions are invited, a fact-based synthesis statement explaining their decision and outlining what is needed to prepare a revision will be listed below. Note: If this manuscript was transferred from JNeurosci and a decision was made to accept the manuscript without peer review, a brief statement to this effect will instead be what is listed below.

Based on your replies to your reviews with J. Neuro, I have determined that you have satisfatorily addressed the concerns of the reviewers, and that your manuscript is therefore suitable for publication in eNeuro.
